# Ultrasound-assisted synthesis of pyrimidines and their fused derivatives: A review

**DOI:** 10.1016/j.ultsonch.2021.105683

**Published:** 2021-07-27

**Authors:** Mateus Mittersteiner, Fellipe F.S. Farias, Helio G. Bonacorso, Marcos A.P. Martins, Nilo Zanatta

**Affiliations:** Núcleo de Química de Heterociclos (NUQUIMHE), Departamento de Química, Universidade Federal de Santa Maria, 97105-900 Santa Maria, Brazil

**Keywords:** Pyrimidines, Ultrasound, Synergic effects, Heterocycles, Catalysts

## Abstract

The pyrimidine scaffold is present in many bioactive drugs; therefore, efficient synthetic routes that provide shorter reaction times, higher yields, and site-selective reactions are constantly being sought. Ultrasound (US) irradiation has emerged as an alternative energy source in the synthesis of these heterocyclic scaffolds, and over the last ten years there has been a significant increase in the number of publications mentioning US in either the construction or derivatization of the pyrimidine core. This review presents a detailed summary (with 140 references) of the effects of US (synergic or not) on the construction and derivatization of the pyrimidine core through classical reactions (e.g., multicomponent, cyclocondensation, cycloaddition, and alkylation reactions). The main points that were taken into consideration are as follows: chemo- and regioselectivity issues, and the results of conventional heating methods compared to US and mechanistic insights that are also presented and discussed for key reactions.

## Highlights

• Ultrasound is a powerful tool in the synthesis of pyrimidines.
• Selectivity in the synthesis of pyrimidines under ultrasound is demonstrated.
• Synergic effects of US combined with other methods are presented.

## Graphical abstract

**Figure:**
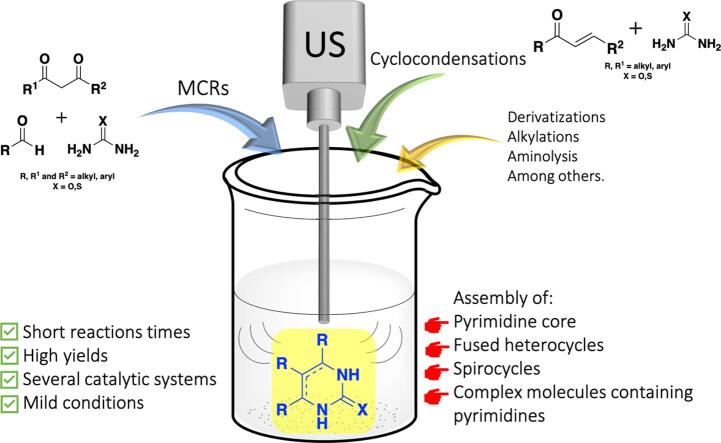

## 1 Introduction

**Fig. 1:**
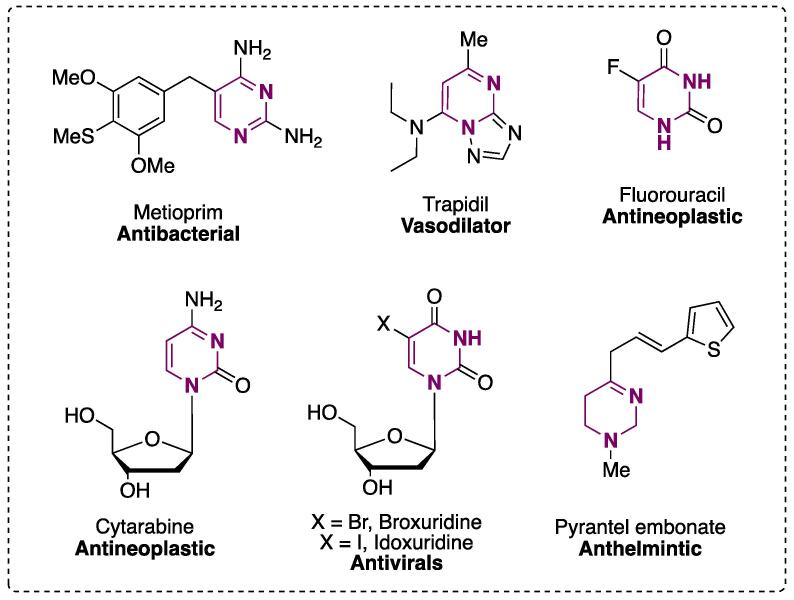
Chemical structures of commercially available drugs containing a pyrimidine ring.

Pyrimidines and pyrimidinones have been used as suitable starting materials for the synthesis of novel scaffolds that are parent to DNA nitrogenated bases (Fig. 1), thus targeting the possible biological and/or pharmacological properties that novel synthesized compounds may present [1]. For example, compounds containing a pyrimidine ring in their structure have been found to act as antiplasmodials [2], as well as caspase [3], hepatitis C [4], [5], NTPDase [6], and cancer [7] inhibitors. Fig. 1 shows some commercially available drugs containing at least one pyrimidine ring (or hydrogenated derivates) in their structure, which shows the wide range of biological activity present in these compounds [8].

Over the last 20 years, several technologies that may assist synthetic organic chemists have emerged as powerful operating tools for heterocyclic synthesis, being the most commonly used as follows: ionic liquids (ILs) as catalysts and/or reaction media [9], [10]; on-grinding methods to promote solvent-free synthesis [11], [12]; and alternative energy sources such as electrosynthesis [13], [14], [15], microwave (MW) [16], [17], and ultrasound (US) irradiation, which has gained special attention in recent years due to its application in organic synthesis [18], [19], [20], [21], [22], [23], [24], [25].

Due to the uniqueness of US in accelerating many organic reactions through cavitation, it provides shorter reaction times and increased yields compared to conventional heating methods or systems involving catalysts [26], [27]. The cavitation phenomenon — which comprises the formation, growth, and collapse of bubbles irradiated with sound — produces enormous amounts of energy, and converts kinetic energy into heating spots [20], [28]. Several US-based techniques have been developed in the last few years, with the aim being to take advantage of the synergic effects that may be provided by US combined with other components, such as ILs [29], [30], [31], pyrimidine-based [32] and other catalysts, [33] and MW irradiation [34].

When working with heterocyclic synthesis, one very important issue is the regioselectivity of the obtained compounds, especially in cyclocondensation reactions [35], [36]. The selectivity of these reactions is mostly due to the reaction conditions, such as solvent choice and temperature [37], the use of additives like BF_3_(OEt)_2_, H_2_SO_4_, and HCl to promote selectivity [38], [39], [40], [41], and steric effects that favor the synthesis of only one regioisomer [42]. Since reactions conducted in US are faster than conventional heating, one may imagine that the short reaction time is not enough to selectively prepare one isomer as a sole product; however, a comparative study showed that the selectivity in obtaining regioisomerically pure pyrazoles leads to only one isomer being furnished, under both conventional heating and US, which indicates that the selectivity is not affected by the US [43].

**Fig. 2:**
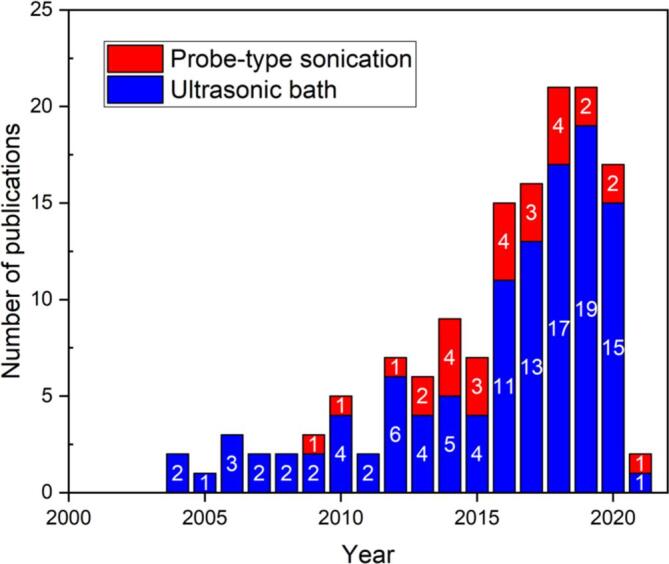
Publications with the terms “ultrasound”, “pyrimidine”, and “synthesis”, found in databases covering the period between 2000 and February 2021.

The relevance of the pyrimidine core and the advantages of using US provide a powerful tool to prepare these six-membered nitrogenated scaffolds. In recent years there has been constant growth in the use of US to prepare several pyrimidine-based heterocycles. Fig. 2 shows this evolution from 2000 onward, reflected by the increasing progression in the number of publications (there was only one article prior to 2000 — from 1987 [44] to be precise). The term “ultrasound pyrimidine synthesis” was used as search parameter in the Web of Science and Scifinder databases, which resulted in a total of 140 items up to February 2021. Among these, 112 (about 80% of the references in this work) applied methodologies that employ the use of ultrasonic bath, while 28 (20%) used probe-type sonication (Fig. 2). The application of probe-type methodologies in pyrimidine synthesis started in 2009 [45], however, up to this date, ultrasonic bath is still the most commonly used methodology.

Probe-type systems have high cavitational intensity in contrast to low operating volumes [46]. Acoustic energy is introduced directly into the liquid and the power dissipated in the reaction mixture can normally be changed, although the frequency of irradiation, in most cases, remains constant. The probe diameter and the height of the liquid in the reactor and other parameters, control cavitational activity and need to be properly selected at suitable levels depending on the application [46]. Despite this, the ultrasonic bath remains the most widely available and cheapest source of ultrasonic irradiation [46], [47], which is perhaps the main reason for being still so widely used. The parameters in the latter are not so precisely adjusted, and cavitation occurs indirectly, thus, reducing the number of variables that can influence the outcome of the reactions [47].

Although some reviews regarding the synthesis of heterocycles performed in US are available in the literature [19], [20], [23], [24], [26], [48], no comprehensive reviews considering US-assisted synthesis of this important heterocyclic scaffold were found, and considering the increasing number of publications in the last few years (140 references in total, with 126 since 2010), a literature review is necessary to summarize the achievements made, which will assist synthetic chemists aiming for new reactions and applications in those already established. For better understanding, this review is divided according to the type of reaction used to prepare the final pyrimidine scaffold, that is: *i)* multicomponent reactions (e.g. Biginelli reaction) for the synthesis of di- and tetrahydropyrimidines and fused derivatives, *ii)* cyclocondensation reactions of α,β-unsaturated ketones with *NCN*-dinucleophiles, *iii)* miscellaneous reactions, and *iv)* derivatizations on the pyrimidine ring.

## 2 Synthesis of pyrimidines via multicomponent reactions (MCR)

### 2.1 MCR in the synthesis of non-fused pyrimidines and pyrimidinone scaffolds

The Biginelli reaction is a three-component reaction between an aldehyde, a 1,3-dicarbonylic compound (or its equivalent), and a (thio)urea derivative that has been used to successfully assemble 3,4-dihydropyrimidin-2(1*H*)-ones [49], [50]. In fact, the great success of this MCR has made it an important item in undergraduate experimental organic chemistry classes for introducing the concept of MCRs [51]. Since its discovery, several catalytic and enantioselective routes have been proposed, with the aim of improving yields, lowering reaction times, and preparing enantiopure products [52], [53], [54], [55], [56].

**Scheme 1:**
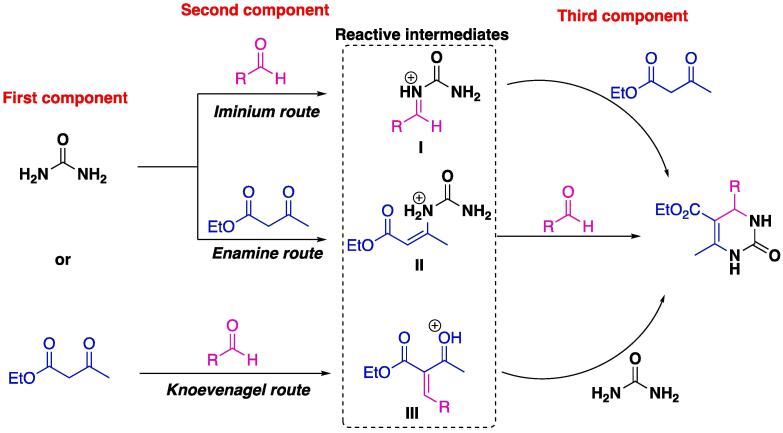
Three possible routes for the Biginelli MCR. Adapted with permission from Nagarajaiah, Mukhopadhyay, and Moorthy (2016) [50] and Puripat et al. (2015) [54]. Copyright (2021) American Chemical Society.

The mechanism of the Biginelli reaction is a topic that has been under investigation for years [57], [58], [59], since three main pathways that lead to the same product are possible when using acidic media (Scheme 1). When urea is the first component and aldehyde the second, the reactive intermediate **I** (iminium ion) is obtained through nucleophilic addition, whereas when the 1,3-dicarbonyl compound (ethyl acetoacetate) is the second component, the 1,4-conjugated Michael-type protonated adduct **II** is obtained. The third route is based on the Knoevenagel condensation between the 1,3-dicarbonyl compound and the aldehyde, which furnishes protonated **III**. The latest achievement in elucidating its mechanism is through artificial force-induced reaction calculations, in which it was found that the iminium route (reactive intermediate **I**) is the most favored, followed by the addition of the 1,3-dicarbonyl compound. It was also found that a second urea molecule catalyzes nearly every step of the process, and protic and aprotic solvents furnish identical results [54]. ESI(+)-MS experiments show that the Knoevenagel route is too slow and does not likely significantly contribute to the synthesis of the Biginelli adduct. Only one intermediate in agreement with the enamine route was detected, whereas several intermediates associated with the iminium route were detected, thus indicating it to be the most feasible route for the Biginelli MCR [60].

**Scheme 2:**
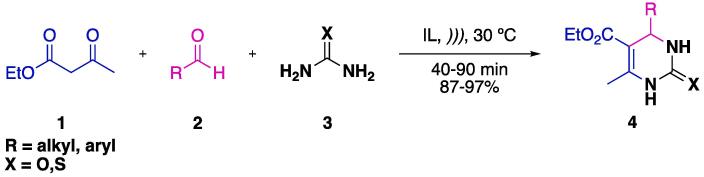
US/IL catalytic system for the Biginelli MCR, reported by Srinivasan et al. (2004) [62].

Given that the Biginelli reaction requires catalysts (usually acidic media) and harsh reaction conditions (other catalytic additives and high temperatures) [61], the use of US irradiation has become an interesting source of alternative energy for this reaction. The first report on the application of US in this MCR relied on the application of ILs as solvent and catalysts (instead of the usual mineral acid) combined with US irradiation (Scheme 2) [62]. Given the proven efficiency of ILs, since their discovery, as both solvents and catalysts in a wide range of organic reactions [63], [64], [65], the combination of ILs with US may result in a very efficient catalytic system for the Biginelli reaction (among others). The authors initially evaluated the reaction between β-ketoester **1** with an aldehyde **2** and (thio)urea **3** to furnish 3,4-dihydropyrimidin-2(1*H*)-one **4**. Different ILs derived from 1,3-dibutylimidazolium (BBIM) and 1-butylimidazolium (HBIM) with different anions (Br^-^, Cl^-^, ClO_4_^-^, BF_4_^-^, and PF_6_^-^) were used. The optimal condition was chosen based upon the time for complete conversion and isolated yield of the final product ([HBIM]BF_4_, 45 min, 95% yield). Having defined the best reaction conditions, the reaction scope was explored, and yields above 87% were obtained in all cases (both electron-withdrawing and electron-donating groups). Remarkably, aldehydes bearing strong electron-withdrawing groups (e.g., 4-NO_2_, 2-F, and 2-Br) required longer reaction times (25, 15, and 15 min more, respectively) [62].

Control reactions were conducted in order to verify possible synergic effects. No reaction was observed when using US combined with molecular solvents (MeCN, EtOH, THF, and CH_2_Cl_2_) in the absence of IL. And when the reaction was conducted under conventional conditions (stirring at 30 °C without US irradiation), once again, no formation of the product was detected, which indicates a synergic effect of the US/IL combined system [62].

**Fig. 3:**
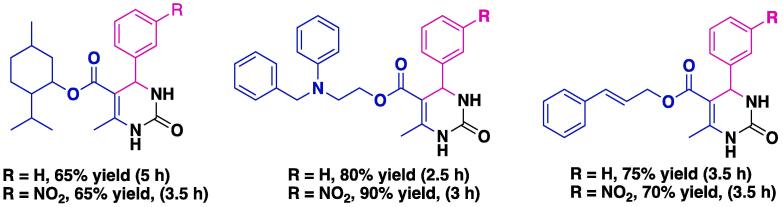
Chemical structures, isolated yields, and reaction times required to prepare novel 3,4-dihydropyrimidin-2(1*H*)-ones using the US/NH_4_Cl catalytic system reported by Stefani et al. (2006) [66].

Another catalytic system reported for the Biginelli MCR is US/NH_4_Cl, using MeOH as solvent [66]. Using this method, eight novel 3,4-dihydropyrimidin-2(1*H*)-ones **4** were prepared at moderate to high yields (65–90%, Fig. 3). All the newly synthesized derivatives were tested for their antioxidant activity, and, notably, compounds bearing the β-aminoester moiety exhibited strong activity against lipid peroxidation induced by iron and EDTA, and when R = H, reduction of reactive oxygen species was also observed [66].

There have been several reports in which the Biginelli MCR is explored using US as the energy source, with slight changes in the reaction conditions (e.g., solvent, catalyst, or additive used — see Table 1). Besides the classical Biginelli MCR (β-ketoester, aldehyde, and (thio)urea), modifications have been made throughout the years to attain the synthesis of the pyrimidine core via MCR, but using different starting materials (ketones, malononitrile, etc.). This demonstrates the wide range of substrates tolerated for the assembly of the pyrimidine core through MCR, as well as the preparation of the final scaffolds with unique substitution patterns (Table 1). Generally, the authors sought optimization of the reaction conditions proposed, and, under the optimal conditions, a scope varying the substituents in at least two of the three starting materials was pursued. The results are often presented in terms of comparison with conventional methods (e.g., heating) and, in some cases, with methods using MW-assisted synthesis.

US irradiation also acts synergistically when combined with other chemicals and/or materials (commonly used as catalysts); for example, the synergetic effect produced from US combined with ILs (Entry 10 in Table 1), in which the corresponding pyrimidines were not obtained when the reaction was conducted in molecular solvents or without US irradiation [62]. Likewise, the same synergetic effect was observed between US and mesoporous Santa Barbara Amorphous (SBA) silica [67], [68]. SBA may have different surfaces that can be functionalized by acidic, basic, or metallic means, which makes it a promising candidate in the development of environmentally friendly synthetic methods [68], [69]. Sulfonic acid covalently functionalized in SBA under US irradiation works as a catalyst, and, compared to other methods, gives high yields in the synthesis of heterocycles (pyrimidine-, pyridine-, and imidazole-based) [67]. The data regarding the synthesis of the tetrahydropyrimidines-5-carboxylate scaffold are presented in Table 1 (Entry 13).

**Table 1:** Conditions used for the MCR assisted by US to assemble the pyrimidine core.^a^ ([70], [71], [72], [73], [74], [75], [76], [77], [78], [79], [80], [81], [82], [83], [84], [85], [86], [87], [88], [89], [90].)




a R, R^1^ and R^2^ = alkyl and/or aryl substituents.

As a general remark, when comparing different methods (conventional heating, MW, and US), the isolated yields of the final compounds are higher and obtained at greater purity when US is used. Besides this, the reaction time is greatly diminished (in most cases, from hours to minutes). For instance, in Table 1, entries 10 – 16 present the reaction of a β-ketoester, an aldehyde and (thio)urea. Several catalysts such as ILs, graphene oxide, acid- and metal- based catalysts were used. The yield of the product was very similar on all the methods applied (up to 98%), however, the reaction time was greatly affected, varying from 6 to 90 min, which shows the synergic effect between the acid catalyst used and the US. Thus, current progress strongly indicates that these US-based procedures applied to MCRs that seek pyrimidine scaffolds, are simpler, safer, more environmentally friendly, and less expensive synthetic approaches.

### 2.2 MCR in the synthesis of fused pyrimidines and pyrimidinone scaffolds

Compared to non-fused heterocycles, the ones fused with a pyrimidine core are known to significantly modify several physical and chemical properties (e.g., selectivity, lipophilicity, polarity, and solubility), thus crucially contributing to the design and application of molecules with promising biological activity [91]. Due to them exhibiting a wide range of activity, fused pyrimidines have been extensively pursued in drug design and discovery. Furthermore, their scaffolds are present in essential vitamins such as riboflavin and folic acid [91], [92].

**Fig. 4:**
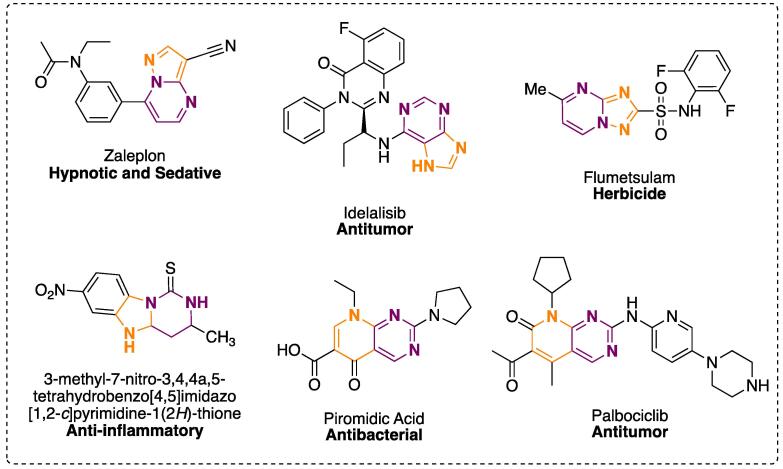
Some pyrimidine-fused compounds and their biological features.

Fused pyrimidines have been observed to have antitumor [93], [94], antibacterial [91], antihyperlipidemic [95], anti-inflammatory [96], herbicidal [97], [98], hypnotic, and sedative [99] properties (Fig. 4). Besides their applications in biology, pharmacology, and medicinal chemistry, they are also notable for their use in dyes [100], potential organic semiconductors [101], fluorescent probes for visualization of lipid drops in living cells [102], etc., thus demonstrating the need to develop more efficient synthetic routes for these scaffolds.

The increasing significance of compounds containing fused pyrimidines, as well as their applicability, has similarly promoted advances over the last few years in the methodologies for the synthesis of these compounds [91], [103]. The main method is still the cyclocondensation of β-(di)carbonyl compounds (usually aldehydes and ketones) associated with *NCN*-dinucleophiles (in which one nucleophilic nitrogen is necessarily inside the heterocycle) such as 2-aminoimidazole, 2-aminopyridine, 2-aminooxazoline, 3-amino-1*H*-1,2,4-triazole, etc. [91], [104], [105]. Thus, US irradiation has emerged as a powerful technique for the synthesis of pyrimidine-fused heterocycles (especially azoles), due to promoting shorter reaction times (usually decreasing from hours to minutes) and higher yields compared to conventional methods [106].

**Scheme 3:**
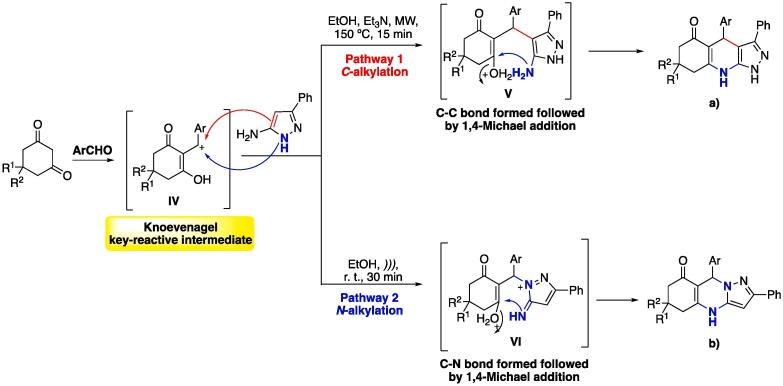
Possible products obtained for the MCR of 5-aminopyrazoles, cyclic aldehydes, and cyclic 1,3-diketones, using either a) MW or b) US irradiation — reported by Chebanov et al. (2008) [108]. Mechanism adapted from Maleki and Aghaei (2017) [112] and Sharma, Vala and Patel (2020) [111], published by The Royal Society of Chemistry.

In relation to MCRs, selectivity is a concern due to the high probability of several potential parallel reaction pathways, which, when not controlled, may lead to complex mixtures of products [107]. US irradiation has been shown to act as a mild catalyst for achieving a single isomer instead of multiple ones, playing an important role in the regio- and chemoselectivity of MCRs [108], [109], [110], [111]. For instance, different products could be obtained (Scheme 3), even when starting from the same building blocks (5-aminopyrazoles, aromatic aldehydes, and cyclic 1,3-diketones). Initially, the reaction (Scheme 3) occurs on the Knoevenagel adduct (in its keto-enol equilibrium form) to furnish a stabilized carbocation (intermediate **IV**), which can suffer a nucleophilic attack from both nucleophilic NH or the C = C of the aromatic ring (no reaction of the NH_2_ moiety was observed). The selectivity of the reaction was fully controlled in order to form the *C*-alkylated (intermediate **V**) product under MW heating (up to 150 °C), whereas when the reaction was done at r. t. using US as catalyst, only the *N*-alkylated product was obtained (intermediate **VI**). Both intermediates **V** and **VI** suffer an intramolecular Michael addition to furnish **a)** 1,4,6,7,8,9-hexahydro-1*H*-pyrazolo[3,4-*b*]quinolin-5-ones or **b)** 5,6,7,9-tetrahydropyrazolo[5,1-*b*]quinazolin-8-ones — see Scheme 3 [108], [111], [112]. Both procedures usually result in pure products (that precipitate out of the solution), without requiring further purification [108]. The selectivity observed is mainly attributed to the temperature, as observed in other experiments under conventional heating. Also, additives (e.g. a tertiary amine such as *N*-methylmorpholine or triethylamine) can be added to improve the yield [108]. Thus, a divergent protocol was developed depending on the energy source used.

The reports that address the use of heterocyclic amidines as nucleophiles, and which seek the synthesis of pyrimidine-fused scaffolds, generally *a)* optimize the reaction conditions, *b)* perform scope assessment, and *c)* present studies of the reaction times and isolated yields compared to other methods such as conventional heating or MW irradiation. Table 2 summarizes these reactions for different heterocycles that can be fused to the pyrimidine core by using US irradiation.

**Table 2:** Conditions used for the MCR assisted by US, for assembling fused-pyrimidine and pyrimidinone scaffolds.a. ([113], [114], [116], [117], [118], [119], [120], [121], [122], [123], [124], [125], [126], [127].)




a R, R^1^ and R^2^ = alkyl and/or aryl substituents. ^b^ Four-component reaction.

### 2.3 MCR in the synthesis of fused pyrimidines, using readily available pyrimidine scaffolds

In addition to MCR being used in the formation of the pyrimidine core, these reactions are also widely explored with readily available pyrimidine-containing scaffold reagents (commercially available or previously synthesized) in the formation of fused, non-fused, and spiro compounds (Table 3, Table 4). These pyrimidine-based starting materials and their derivatives have also been explored in several synthetic protocols, and due to them already possessing a formed heterocycle, they act as building blocks for the synthesis of more complex molecules that are useful in medicinal chemistry and materials science [128], [129], [130].

**Table 3:** Conditions used for the MCRs assisted by US, for readily available pyrimidine core based reagents.a. ([132], [133], [134], [135], [136], [137], [138], [139], [140], [141], [142], [143], [144], [145], [146], [147], [148], [149], [150], [151], [152], [154], [155], [156], [157], [158].)




a R,R^1^ and R^2^ = alkyl and/or aryl substituents. ^b^ Four-component reaction.

The use of previously formed pyrimidine-based reagents presents some very interesting advantages; for example: *a)* reducing the reaction steps and the generation of by-products, since fewer parallel reactions occur and there are fewer reactive centers in competition; *b)* allowing the design of more complex structures (with more functional groups), since the focus is on other parts of the molecule rather than assembling the pyrimidine core and, therefore, it is possible to readily form fused heterocycles, non-fused heterocycles, spiro compounds, etc.; and *c)* furnishing a highly versatile starting material with multiple reaction sites, which enables the synthesis of different derivatives of these scaffolds (*N-*, *O-*, *S-*, and C=C nucleophilic centers are often present), thus making the incorporation of the entire pyrimidine nucleus a very interesting synthetic strategy, from both a biological and industrial point of view, given that this can result in interesting compounds with enhanced properties [110], [129], [131].

These enhanced properties of the products obtained from the use of readily available pyrimidine-based reagents motivated synthetic chemists to explore their chemistry (in terms of reactivity and selectivity), and more recently, especially in the past three years, these starting materials have joined the constant growth of MCRs along with the use of US radiation. These reactions are described in Table 3. Of note is the pursuit of more eco-friendly, low-cost additives and catalysts with high recyclability and milder reaction conditions. It is worth highlighting the use of water and ethanol as solvents, as well as solvent-free and catalyst-free reactions. The efficiency of US in these MCRs can be seen in the reaction yields — which are mostly good to excellent — and the vast range of products achieved, in short reaction times (from minutes to hours) and under milder and more eco-friendly conditions.

### 2.4 MCR in the synthesis of pyrimidines containing spirocycles

Spiro-based heterocyclic systems — that is, a quaternary carbon atom common to two rings (hetero- or carbocycles) — are promising compounds in several areas; for example, pharmacology, crystallography, materials science, biochemistry, molecular biology, and engineering [159], [160], [161], [162], [163], [164], to name just a few. Besides their unique molecular characteristics related to stereochemistry, the great interest in spiro compounds is filled by an extremely wide range of the aforementioned useful properties [24], [110]. With this in mind, compounds joining these two structures (spirocycles and the pyrimidine core) furnish appealing final compounds [129].

MCRs performed under US irradiation have emerged as a valuable improvement for the synthesis of spirocycles, due to decreasing the number of reaction steps and reaction time and increasing yields, among other advantages [165]. The use of US together with pyrimidine-containing reagents to obtain spiro compounds dates from a few years ago — it occurred for the first time in 2009 when, serendipitously, spiro compounds (instead of the expected products) were obtained under US irradiation [110].

**Scheme 4:**
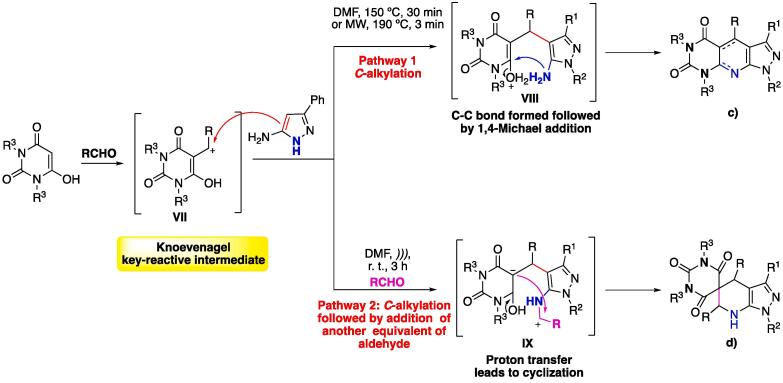
Selectivity of the MCR of 5-aminopyrazoles, aldehydes, and barbituric acids reported by Muravyova et al. (2009) [110]. Mechanism adapted from Kruithof, Ruijter and Orru (2011) [166] and Maleki and Aghaei (2017) [112].

When using barbituric acids as starting materials, temperature was reported as the directing factor in the MCR, since the formation of the expected heterocycle was not observed as a sole product, instead either fused or spiro-compounds were obtained (Scheme 4 **c)** and **d)**, respectively). The proposed mechanism (Scheme 4) initially involves the formation of the Knoevenagel adduct (Intermediate **VII**), for both pathways. To achieve the expected fused heterocycle, which goes through the initial C–C bond formation followed by intramolecular 1,4-Michael addition, the reactions were done at higher temperatures (~150–190 °C), using conventional methods or MW conditions, which leads to the Hantzsch-type product pyrazolo[4′,3′:5,6]pyrido[2,3-*d*]pyrimidin-5-ones shown in Scheme 4 **c)**. On the other hand, when the reaction is performed under US irradiation at r. t., the amino moiety of the Knoevenagel adduct reacts with a second portion of the aromatic aldehyde to furnish an imine intermediate, which, through proton transfer (from the α-carbonyl position to the α-imine position, intermediate **IX**), furnishes a very reactive electrophilic center, which, in turn, undergoes cyclization with the negative charge to furnish the 1,4,6,7-tetrahydro-1′*H*-spiro[pyrazolo[3,4-*b*]pyridine-5,5′-pyrimidine]-2′,4′,6′(3′*H*)-trione shown in Scheme 4 **d)** [110], [112], [166]. The proposed mechanism is supported by quantum mechanics calculations (DFT – B3LYP) [166], thus demonstrating that US irradiation acts directly on the chemoselectivity of the reaction.

A range of spiro compounds have already been obtained from readily available pyrimidine-containing reagents (e.g. barbituric acid) under US radiation (Table 4), including spiro-(oxy)indoles [167], [168], [169], spiro-pyrans [153], spiro-naphthoquinolines [170], and spiro-indazolophthalazines [171], with a wide variety of reaction conditions free from catalysts (entries 1–4 in Table 4), but with the presence of promising catalysts such as IL/MOFs (Entries 10 and 11) [172], [173]. Table 4 summarizes the spiro compounds — obtained up to the present time and with their respective US conditions and yields.

**Table 4:** Conditions used for the MCR in the assembly of spiro compounds assisted by US.a. ([174], [249], [250], [251], [252], [253], [254].)




a R,R^1^ and R^2^ = alkyl and/or aryl substituents.

## 3 **Cyclocondensation reactions of** α**,**β**-unsaturated ketones with *NCN*-dinucleophiles**

α,β-Unsaturated ketones (also known as enones) are readily available *CCC*-building blocks that have many uses in heterocyclic chemistry, due to the enhanced electrophilicity of the β-carbon over the carbonyl carbon [175], [176], [177]. Thus, using *NCN*-dinucleophiles, one can assemble several pyrimidine-based heterocycles through [3 + 3] cyclocondensation reactions [40], [178]. Although efficient, these types of cyclocondensation reactions — especially using poor nucleophiles such as urea derivatives — usually require harsh reaction conditions (long reaction times and high temperatures) [179] and transition-metal catalysts or acidic media to enhance the electrophilicity of the carbonyl carbon of the enone [41], [180]. Thus, US irradiation emerged as an efficient alternative route for performing these [3 + 3] cyclocondensation reactions.

**Scheme 5:**
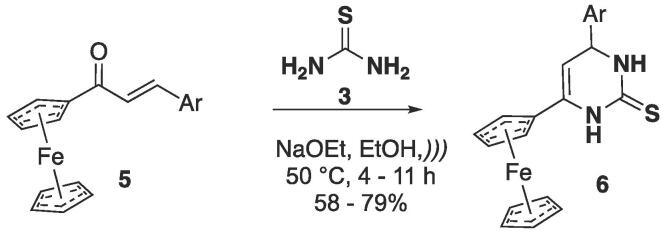
Synthesis of ferrocene-based pyrimidine-2(1*H*)-thiones **6**, reported by Toma, Putala and Salisová (1987) [44].

One of the first reports about the use of enones was the cyclocondensation reaction between ferrocene-chalcone **5** based derivatives and thiourea **3** in basic media, which furnished ferrocene-containing pyrimidine-2(1*H*)-thiones (Scheme 5) [44]. Using sodium ethoxide as base, the reaction was done in ethanol at 50 °C and for the optimal time determined by thin layer chromatography (TLC), which varied according to the substituent in the starting **5** (electron-withdrawing groups as 4-Cl required longer reaction times).

**Scheme 6:**
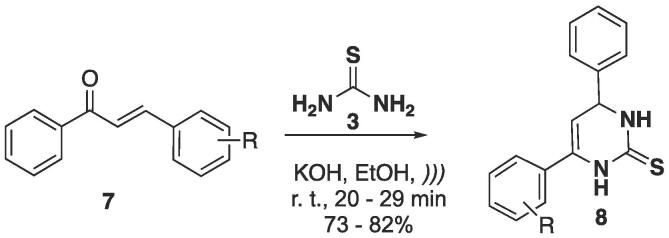
Synthesis of 6-aryl-4-phenyl-3,4-dihydropyrimidine-2(1*H*)-thiones **8** using chalcones **7**, reported by Safaei-Ghomi and Ghasemzadeh (2011) [181].

Chalcone derivatives were also used in the cyclocondensation with thiourea **3**, using KOH as base and EtOH as solvent, and the reaction was irradiated with US for 20–29 min (depending on the substituent in the starting **7**). The products were obtained at 73–82% yields (Scheme 6) [181].

The authors also did a comparative study between conventional heating (oil bath) and US. The results were compared in terms of the time until there was no more consumption of starting material detected by TLC analysis, as well as the isolated yields of the final compounds — see Table 5. The reaction times decreased from 5.5 to 6.5 h to 0.4–0.5 h, which, in most cases, is ten times faster. The lower yield obtained under conventional heating (R = 3-Me, 54%) rose to 80% under US. It is important to mention that no strongly electron-withdrawing group (nitro, bromo, chloro, etc.) was evaluated, thus, this could strongly affect both reaction time and yield [181].

**Table 5:** Comparative study of the reaction times and isolated yields of pyrimidine-2(1*H*)-thiones **8**[181].

R	Conventional conditions		US irradiation	
	Time (h)	Yield (%)	Time (min)	Yield (%)
H	5.5	65	20	82
2-Me	6.0	55	22	78
3-Me	6.0	54	22	80
4-Me	5.5	58	24	76
2-OMe	6.0	60	26	76
4-OMe	6.0	61	24	75
2,4-OMe	6.5	65	25	73
4-N(Me_2_)	5.5	55	29	75

**Scheme 7:**
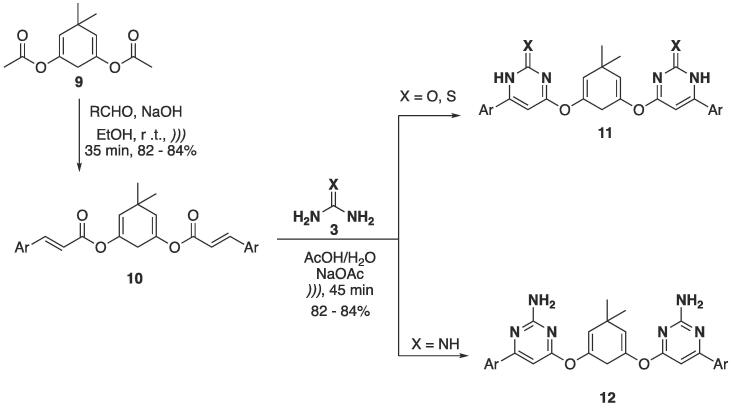
Aldol condensation followed by [3 + 3] cyclocondensation with *NCN*-dinucleophiles to furnish bis-pyrimidine derivatives **11–12**, reported by Dabholkar and Ansari (2012) [182].

A fully US-based strategy was followed to prepare *bis*-pyrimidine derivatives, using the diester **9** as starting material (Scheme 7). Initially, an aldol condensation between the ester and aldehydes in basic media was performed (NaOH, EtOH, *)))*, 35 min), which furnished α,β-unsaturated esters **10** at 82–84% yields. Subsequently, cyclocondensation with *NCN*-dinucleophiles (urea, thiourea, and guanidine) furnished *bis*-pyrimidines **11–12** at 82–84% yields (Scheme 7) [182]. The authors also performed a comparative study using conventional conditions — for the synthesis of **10**, the reaction took 2.5 h to be completed (72–73% yields), compared to 35 min when US was used (82–84% yields). The synthesis of **11–12** was achieved via conventional conditions by stirring for 4 h under refluxing water, or by 45 min of US irradiation (for the latter, a 10% increase in yield was observed).

**Scheme 8:**
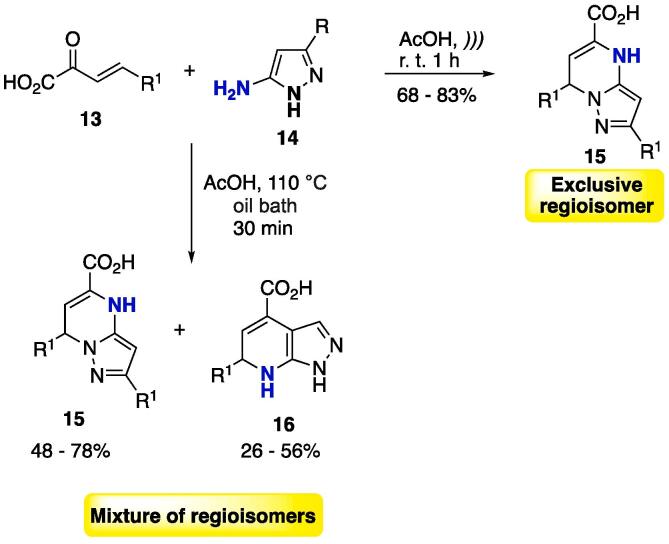
Cyclocondensation reaction between enones **13** and 5-aminopyrazoles **14** as a selective protocol for obtaining a single isomer of pyrazolo[1,5-*a*]pyrimidines, reported by Chebanov, Sakhno and Desenko (2012) [183].

Enones **13** were cyclocondensed with 5-aminopyrazoles **14** (AcOH, *)))*, r. t. 1 h) to furnish one single regioisomer of pyrazolo[1,5-*a*]pyrimidine **15** at moderate to good yields (68–83%, Scheme 8) [183]. It is important to note that the authors had previously reported this same reaction under conventional conditions (using refluxing AcOH), in which a mixture of regioisomers **15** and **16** was obtained (Scheme 8) [184]. It is well known that temperature greatly affects the regioselectivity in the cyclocondensation of enones with non-symmetrical *NCN*-dinucleophiles [98]. Conducting the reaction at r.t. using US furnished one single isomer, thus showing that the development of selective protocols using US is feasible.

**Scheme 9:**
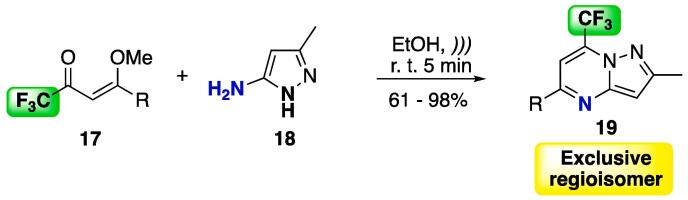
Cyclocondensation reaction between enones **17** and 5-aminopyrazole **18** as a selective protocol for obtaining a single isomer of pyrazolo[1,5-*a*]pyrimidines, reported by Buriol et al. (2013) [185].

The reaction between trifluoromethyl-substituted alkoxy enones **17** and 5-aminopyrazole **18** (Scheme 9) was reported as a selective and fast protocol for obtaining pyrazolo[1,5-*a*]pyrimidines [185]. The reaction was done in EtOH for a period of 5 min (with max. temperature programmed of 75 °C), and the products were obtained at moderate to excellent yields (61–98%).

The authors also performed a comparative study between conventional methods, MW irradiation, and US irradiation for the synthesis of **19** (Table 6). No significant difference was observed for the alkyl substituents at the 4-position of starting enone **17** — in fact, when using US, the yield was slightly (5–6%) lower. However, when aryl substituents were used, the reaction under conventional conditions (oil bath) provided moderate yields in the range of 51–73% (refluxing EtOH, 2 h). When MW was used, the yields improved to 80–93% (EtOH, 5 min), and under the same experimental conditions, US provided 82–96% yields.

**Table 6:** Comparative study of the synthesis of pyrazolo[1,5-*a*]pyrimidines, using oil bath, MW irradiation, and US, reported by Buriol et al. (2013) [185].

R	Isolated yield (%)		
	Oil bath ^a^	Microwave ^b^	Ultrasound ^c^
Me	87	87	82
*i*-Bu	83	84	77
C_6_H_5_	52	80	82
4-F-C_6_H_4_	51	81	96
Naphth-2-yl	73	93	89

Reaction conditions: ^a^ EtOH, 75 °C, 2 h. ^b^ EtOH, MW, 75 °C, 5 min. ^c^ EtOH, US, 68 – 72 °C, 5 min.

**Scheme 10:**
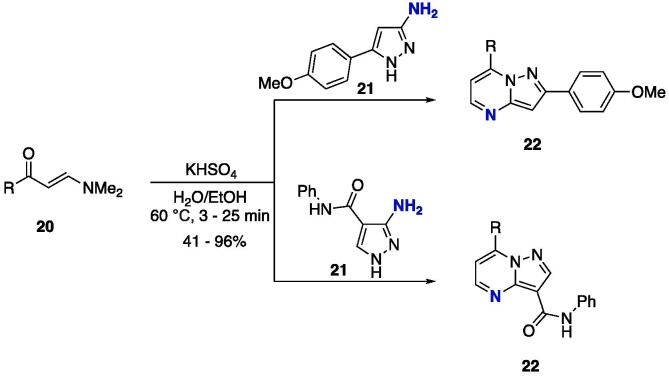
Cyclocondensation reaction between β-enaminone **20** and 3-aminopyrazole **21** to furnish exclusively pyrazolo[1,5-*a*]pyrimidines **22**, reported by Kaping et al. (2016) [188]**.**

The reaction was also extended to the use of β-dimethylaminovinyl ketones **20** as starting materials — which have also been used as efficient building blocks in constructing heterocycles [186], [187] — and 3-aminopyrazoles **21** (Scheme 10). This is an important modification, because the dimethylamino group can lower the electrophilicity of the β-carbon compared to that of the alkoxy moiety of **17**, which was in fact observed, since the authors used KHSO_4_ as catalyst and increased the temperature [188]. The pyrazolo[1,5-*a*]pyrimidines **22** were obtained at moderate to excellent yields (41–95%), and were highly dependent on the structure of the starting β-enaminone **20** [167].

**Scheme 11:**
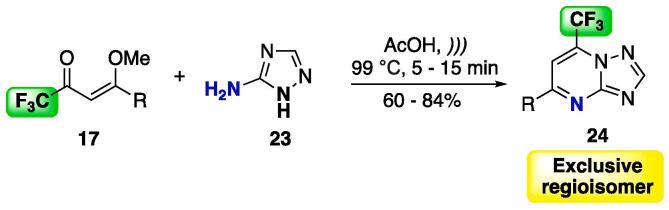
Cyclocondensation reaction between enones **17** and 5-amino-1,2,4-triazole **23**, as a selective protocol for obtaining a single isomer of 1,2,4-triazolo[1,5-*a*]pyrimidines **24**, reported by Frizzo et al. (2014) [106].

Similar to this, the trifluoromethyl-substituted alkoxy enone **17** was reacted with 5-amino-1,2,4-triazole **23** under acidic media to selectively furnish 1,2,4-triazolo[1,5-*a*]pyrimidines (Scheme 11) [106]. During optimization of the reaction conditions, the authors observed that the reaction did not occur when using EtOH or MeCN as solvents or lower temperatures, which suggests lower reactivity of this *NCN*-dinucleophile **24** compared to that of 5-aminopyrazole **18**.

**Scheme 12:**
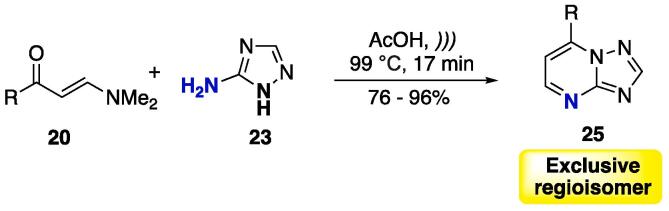
Cyclocondensation reaction between enones **20** and 5-amino-1,2,4-triazole **23**, as a selective protocol for obtaining a single isomer of 1,2,4-triazolo[1,5-*a*]pyrimidines **25**, reported by Frizzo et al. (2014) [106].

The reaction was also extended with the use of β-dimethylaminovinyl ketones **20** as starting materials (Scheme 12), and the 7-substituted 1,2,4-triazolo[1,5-*a*]pyrimidines **25** were obtained at good yields (76– 96%) in one exclusive regioisomer [106].

**Scheme 13:**
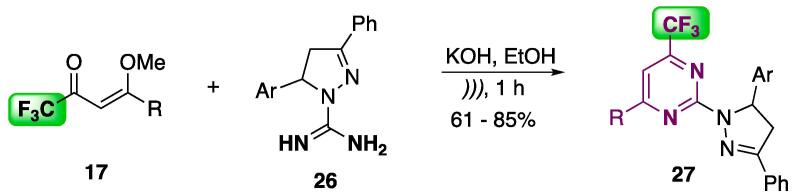
Cyclocondensation of enone **17** and the heterocyclic amidine **26** to furnish 2-pyrazolopyrimidines **27**, reported by Kuhn et al. 2015 [191].

A more environmentally friendly protocol was developed for cyclocondensation of enones **17** and the heterocyclic amidine precursor **26**. The conventional procedure for preparing parent compounds of **27** takes 4–24 h in refluxing MeOH or CHCl_3_ [189]_,_ or requires the use of catalysts such as Ti(O*i*-Pr)_4_ or BF_3_·OEt_2_ [190]. In the present study, the reaction was conducted under US irradiation in the presence of KOH (EtOH, r.t., 1 h, Scheme 13). The 2-pyrazolyl pyrimidines **27** were isolated, at 61–85% yields, by simply filtering the reaction media, with no further purification (recrystallization or chromatography) necessary [191].

**Scheme 14:**
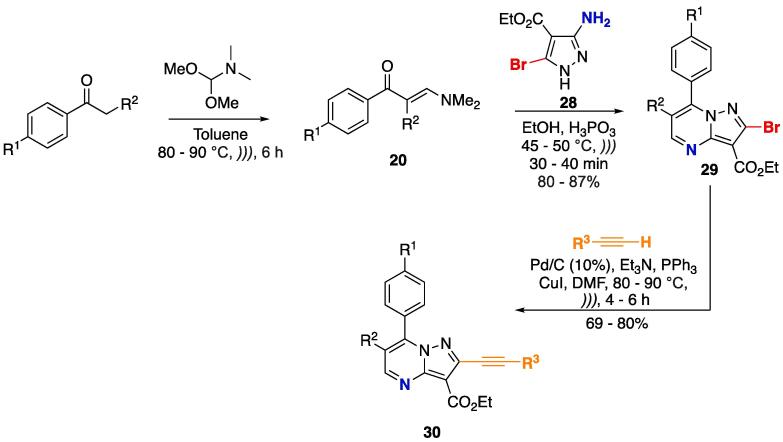
Stepwise US synthesis of **30** via aldol condensation of aryl ketones, followed by cyclocondensation with 3-aminopyrazole **28** and Sonogashira cross-coupling of **29**, reported by Bharath, Rao, and Pal (2017) [192].

The synthesis of 2-alkynyl pyrazolo[1,5-*a*]pyrimidines **30** (Scheme 14) was done using US in all reaction steps [192]. Initially, the known aldol condensation of aryl ketones with *N*,*N*-dimethylformamide dimethylacetal was done (toluene, 80–90 °C, 6 h) to obtain β-enaminones **20**, which underwent cyclocondensation with 3-amino-5-bromopyrazole **28** (EtOH, H_3_PO_3_, 45–50 °C, 30–40 min) to furnish pyrazolo[1,5-*a*]pyrimidines **29** at good yields (80– 87%). The bromine moiety was reacted with terminal alkynes via palladium-catalyzed cross-coupling reaction (Sonogashira-type) to furnish 2-alkynyl pyrazolo[1,5-*a*]pyrimidines **30** at good yields (69–80%) in short reaction times (4–6 h) compared to those of conventional methods (18–24 h, depending on the substrates) [193], [194], [195].

**Scheme 15:**
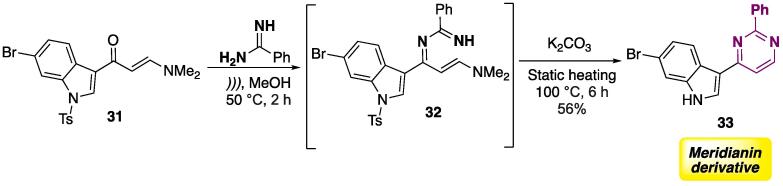
Synthesis of Meridianin derivative **33**, using a hybrid US-static heating technique, reported by Jiang et al. (2018) [197]**.**

The synthesis of Meridianin derivatives — which are marine alkaloids isolated from *Aplidium meridianum* [196] — was done using a hybrid US-static heating technique (Scheme 15) [197]**.** Initially, indolyl-β-enaminone **31** was reacted with benzamidine to furnish **32** (which was not isolated), which was then subjected to static heating to allow cyclocondensation and removal of the tosyl group. Meridianin derivative **33** was isolated at a 56% yield. [197]

**Scheme 16:**
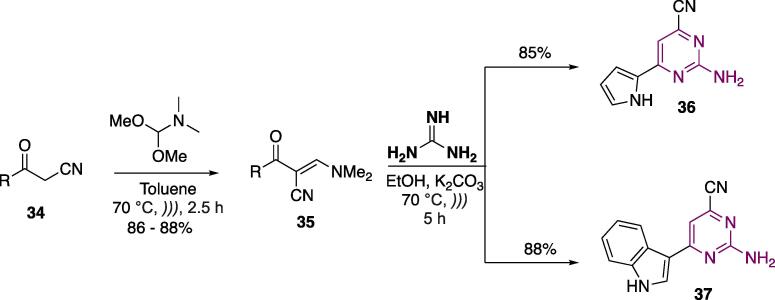
Synthesis of 6-azolyl-2-amino-4-cyanopyrimidines **36** and **37**, reported by Al-Zaydi et al. (2017) [198]**.**

The synthesis of pyrimidines conjugated with 1*H*-pyrrole and 1*H*-indole cores was done using α-cyano ketones **34** as starting materials (Scheme 16). These were reacted with *N*,*N*-dimethylformamide dimethylacetal (toluene, 70 °C, *))),* 2.5 h) to furnish α-cyano-β-enaminones **35** at 86–88% yields. These were then cyclocondensed with guanidine (EtOH, K_2_CO_3_, 70 °C, *))),* 5 h) to furnish 6-azolyl-2-amino-4-cyanopyrimidines **36** and **37** at 85–88% yields (Scheme 16) [198]**.**

**Scheme 17:**
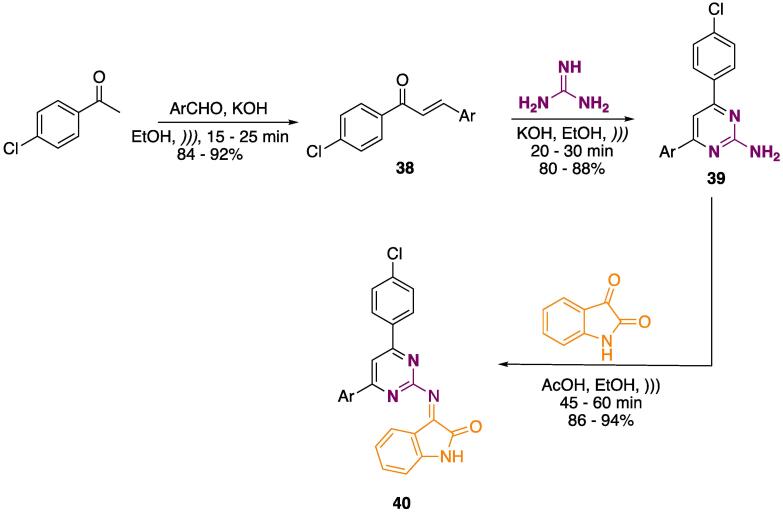
Synthesis of indolin-2-one coupled pyrimidines **40**, reported by Nikalje et al. (2018) [199].

The synthesis of indolin-2-ones coupled to 2-amino pyrimidines was done in a multistep reaction (Scheme 17) [199]. Starting from the condensation of 4-chloroacetophenone with aryl aldehydes (KOH, EtOH, *)))*, 15–25 min), enones **38** were obtained at 84–92% yields. Further cyclocondensation with guanidine (KOH, EtOH, *)))* 20–30 min) furnished 2-aminopyrimidines **39** at 80–88% yields. Lastly, nucleophilic addition using indoline-2,3-dione was performed (AcOH, EtOH, *))),* 45–60 min), and the final products **40** were obtained at 86–94% yields (Scheme 17) [199].

It is important to mention that the authors performed a comparison between US and conventional heating in all reaction steps. When preparing **38**, the reaction time decreased from 240 to 360 min to 15–25 min, and the yields increased from 58 to 78% to 88–92%. In the case of 2-aminopyrimidine **39**, the reaction time decreased from 240 to 360 min to 20–30 min, and the yields increased from 55 to 70% to 80–88%. The nucleophilic addition step led to the time decreasing from 510 to 630 min to 45–60 min, and the yields increasing from 58 to 74% to 85–94% [199]**.**

**Scheme 18:**
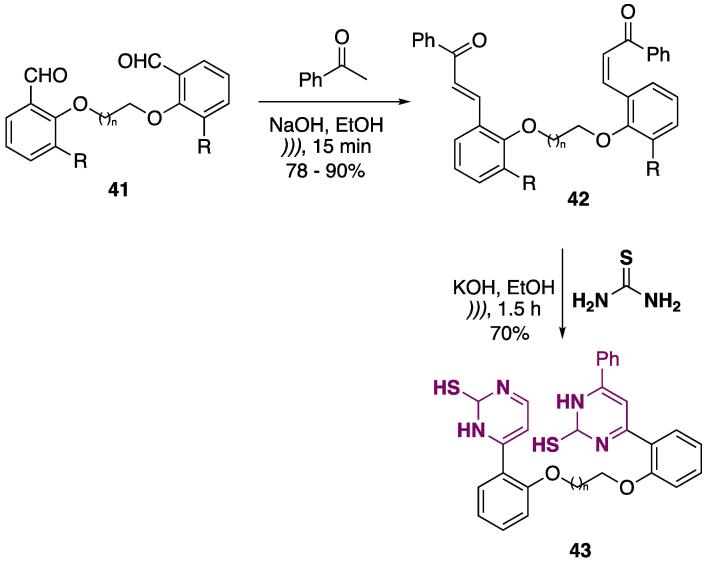
Synthesis of *bis*-pyrimidine **43** using 2-alkoxy benzaldehydes **41** as starting materials, reported by Banaei, Salmanpour, and Karimi (2017) [200].

The synthesis of *bis*-chalcones was done using symmetrical 2-alkoxy benzaldehydes **41** as starting materials (Scheme 18). These were condensed with acetophenone (NaOH, EtOH, *))),* 15 min), and *bis*-chalcones **42** were obtained at 78–90% yields. Further cyclocondensation of **42** with thiourea furnished *bis*-pyrimidine **43** at 70% yield (Scheme 18) [200]**.**

## 4 Miscellaneous reactions

**Scheme 19:**
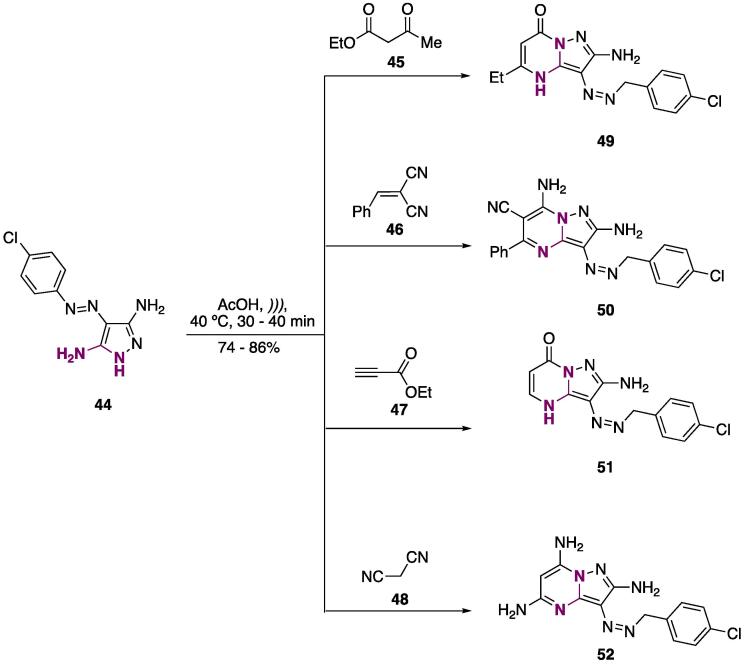
Synthesis of pyrimidines **49–52**, using pyrazole **44** as *NCN*-dinucleophile, reported by Al-Zaydi (2009) [201].

Pyrazole **44** was used as a model to promote cyclocondensation with several electrophiles (**45–48**, Scheme 19), and pyrimidines **49–52** were prepared (AcOH, *))),* 40 °C, 30–40 min) at good yields (74–86%) [201]. A comparative study between conventional heating, MW irradiation, and US irradiation was conducted, and it was found that for these substrates, MW was superior to US (reaction times of 2–5 min for MW and 30–40 min for US). On looking at these results, one can closely relate to the temperature in the reaction vessel, since the authors measured 105 – 110 °C for the flask submitted to MW and 35 – 40 °C for the US one. It is well known that temperature is a key factor in cyclocondensation reactions, thus, the lower temperature that the reaction was conducted under for the US may explain the longer reaction times and lower yields compared to MW [201].

**Scheme 20:**
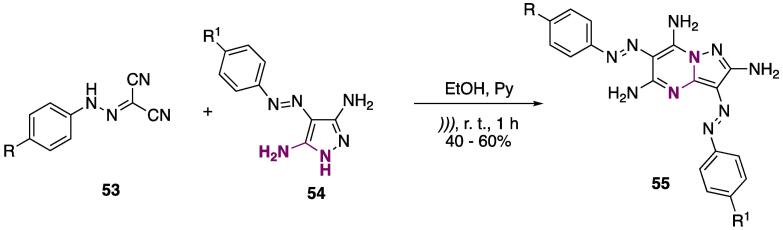
Synthesis of diazo-containing pyrimidines **55**, using 2-arylazomalononitriles **53**, reported by Fouda et al. (2019) [202].

The synthesis of diazo-containing pyrazolo[1,5-*a*]pyrimidines **55** was achieved by reacting 2-arylazomalononitriles **53** with fluorine-containing pyrazoles **54** (R^1^ = F, CF_3_) to furnish **55** at 40–60% yields (EtOH, Py, r.t., *)))*, 1 h) [202]. The authors performed a comparative study, but the observations were the same as the work above: MW irradiation furnished better yields and shorter reaction times than US; however, the reaction done under MW was at 140 °C, while under US it was at r. t. (Scheme 20) [202]**.**

**Scheme 21:**
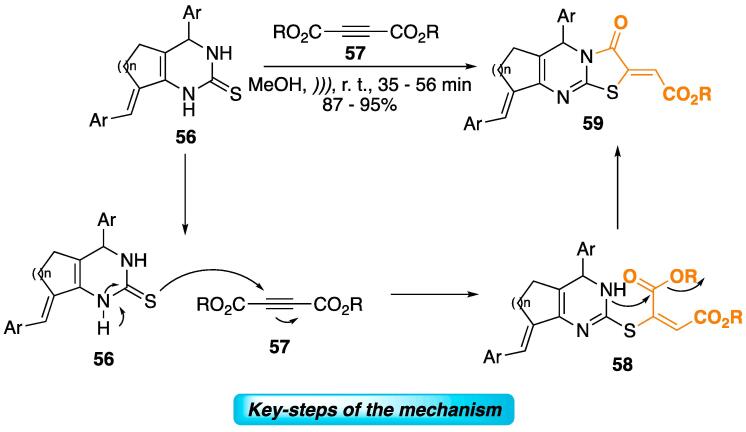
Synthesis of thiazolo[3,2-*a*]pyrimidines **59** from the reaction of pyrimidine-2-thiones **56** and alkynyl esters **57**, reported by Darehkordi, Reentan, and Ramezani (2013) [203].

Pyrimidine-2-thiones **56** were reacted with alkynyl esters **57** to furnish thiazolo[3,2-*a*]pyrimidines **59** at 87–95% yields (MeOH, *)))*, r.t. 35–56 min, Scheme 21) [203]. The mechanism for this transformation is shown in Scheme 21, in which the nucleophilic sulfur attacks the electrophilic carbon of the alkyne through a 1,4-Michael-type addition. In the last step, the ester moiety undergoes aminolysis to furnish **59** [203], [204].

**Scheme 22:**
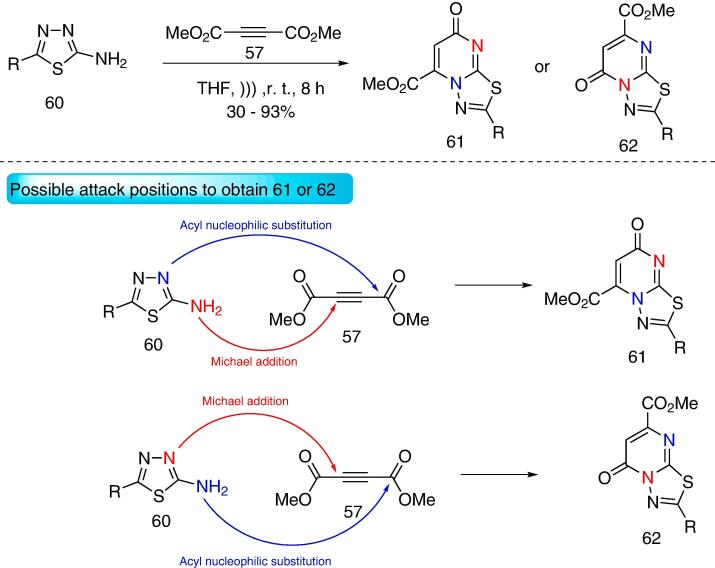
Synthesis of [1], [3], [4]thiadiazolo[3,2-*a*]pyrimidines **61** from 2-aminothiadiazoles **60** and dimethylacetylenedicarboxylate **57**, reported by Dong and Zhao (2019) [205].

By using 2-aminothiadiazoles **60** and dimethylacetylenedicarboxylate **57**, [1,3,4]thiadiazolo[3,2-*a*]pyrimidines **61** were obtained (THF, *)))*, r.t. 8 h) at 30–93% yields (Scheme 22) [205]. Given that **60** are non-symmetrical *NCN*-dinucleophiles, one expects to obtain two isomers (depending on the attacking position of each nucleophilic nitrogen) — either product **61** or **62** (Scheme 22). Interestingly enough, only product **61** was obtained (first pathway). The authors performed DFT calculations to identify the most stable reaction intermediates, and the pathway that furnishes **61** was the less energetic one.

**Scheme 23:**
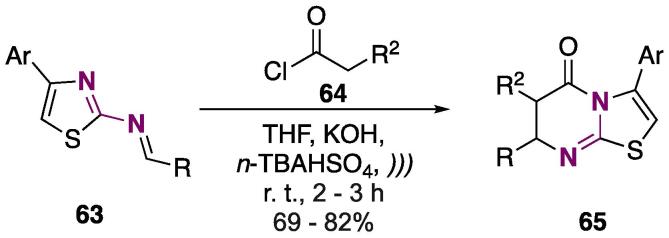
Synthesis of 3,7-diaryl‐6,7‐dihydro-(5*H)*-6-substituted-thiazolo[3,2-*a*]pyrimidin-5‐ones **65**, reported by Gupta et al. (2016) [206].

The synthesis of thiazolo[3,2-*a*]pyrimidin-5‐ones under US irradiation and using a solid–liquid phase transfer catalysis (PTC) — *n*-TBAHSO_4_ — to prepare 3,7-diaryl‐6,7‐dihydro-(5*H*)-6-substituted-thiazolo[3,2-*a*]pyrimidin-5‐ones **65** was reported [206]. The reaction was done using Schiff’s bases **63** and acyl chlorides **64**, through a [4 + 2] cycloaddition reaction (conditions: THF, KOH, *)))*, r.t. 2–3 h — see Scheme 23), and products **65** were obtained at 69–82% yields [206].

PTC is a technique with a green chemistry bias, in which the reaction occurs or is accelerated because of the miscibility of the reagent species [207], [208], [209]. When combined with US, it is expected to further accelerate the reaction, as well as increase conversions for the desired products. The cavitation phenomenon provided by US increases mass transfer around solid particles, as well as the phase boundaries. The use of US is a good technique when supplemented with PTC, given that PTC strongly depends on the transfer between phases [206]. The authors also performed a comparative study for conventional stirring (at r.t.), and they were able to prove the synergism in the US/PTC method, given the higher yields (69–82% and 53–64% with and without US irradiation, respectively) and shorter reaction times (decreasing from 7 to 9 h to 2–3 h) of **65** [206].

**Scheme 24:**
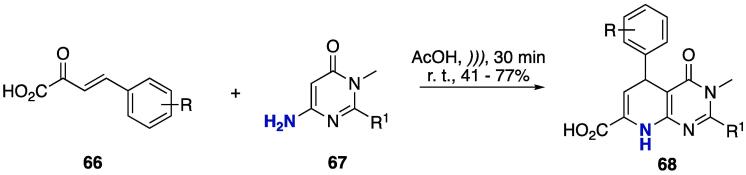
Synthesis of tetrahydropyrido[2,3-*d*]pyrimidines **68** using enones **66** and 6-amino pyrimidines **67**, reported by Quiroga et al. (2016) [210].

The synthesis of tetrahydropyrido[2,3-*d*]pyrimidines **68** was done by using enones **66** and 6-aminopyrimidine **67** (AcOH, *)))*, r.t., 30 min, Scheme 24) [210]. The final products were obtained at 41–77% yields — the yields were highly dependent on the substituent in the aromatic ring of the starting **66**. The reactions were also conducted under MW irradiation (at 110 °C); however, the yields were lower (31–76%) than those obtained with US [210].

**Scheme 25:**
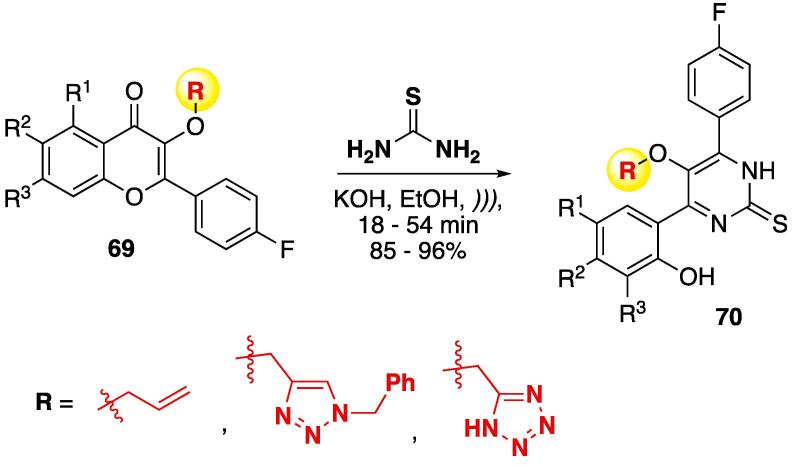
Synthesis of allyl/1,2,3-triazolyl/tetrazol-5-yl containing pyrimidine-2-thiones **70**, through cyclocondensation with thiourea, reported by Dofe et al. (2017–2018) [211], [212], [213].

The synthesis of pyrimidine-2-thiones **70** was done by using 3-alkoxy chromenones **69** and thiourea to furnish allyl/1,2,3-triazolyl/tetrazol-5-yl containing pyrimidine-2-thiones **70**, at good to excellent yields (85–96%, KOH, EtOH, *)))*, 18–54 min — see Scheme 25) [211], [212], [213].

The authors also did a comparative study of the conventional method and US in the synthesis of allyl and 1,2,3-triazolyl derivatives of **70** (Table 7). Regardless the alkoxy moiety, when performed under conventional conditions, the reaction times and isolated yields of the compounds were very similar (190–220 min, 69–79% yields). When US was used, the reaction times decreased to 15–27 min, and yields increased to 87–94%. It is notable that, in both cases, when starting **69** has electron-withdrawing groups (chloro was used as the model), the reaction proceeds faster (entries 2, 5, and 6 in Table 7), but no correlation between structure and the isolated yield of **70** was observed [211], [212].

**Table 7:** Comparative study of conventional method and US in the synthesis of 1,2,3-triazolyl/tetrazol-5-yl containing pyrimidine-2-thiones **70.**

Entry	R^1^	R^2^	R^3^								
				Oil bath		Ultrasound		Oil bath		Ultrasound	
				Time (min)	Yield (%)	Time (min)	Yield (%)	Time (min)	Yield (%)	Time (min)	Yield (%)
1	H	H	H	200	75	24	88	225	69	21	89
2	H	H	Cl	190	78	21	86	195	71	18	91
3	Me	H	H	220	71	27	90	215	68	27	87
4	H	H	Me	230	73	27	93	210	72	24	94
5	Cl	H	Cl	190	78	21	94	190	77	15	93
6	H	Me	Cl	195	74	25	90	205	79	18	91

**Scheme 26:**
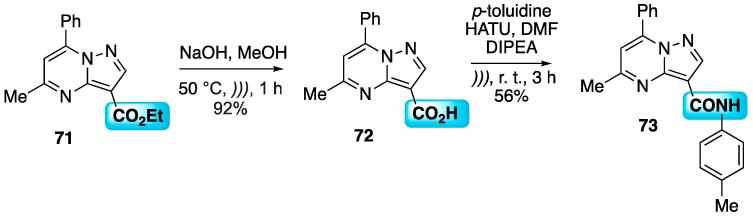
US-assisted hydrolysis followed by HATU-mediated carboxylic acid coupling of pyrazolo[1,5-*a*]pyrimidine **73**, reported by Suresh et al. (2017) [115].

Hydrolysis of the carboxyethyl moiety of pyrazolo[1,5-*a*]pyrimidine **71** was followed by amidation with *p*-toluidine, using hexafluorophosphate azabenzotriazole tetramethyl uronium (HATU) as the coupling agent (Scheme 26) [115]. Initially, the hydrolysis step was done using NaOH/MeOH, at 50 °C under US for 1 h. The carboxylic acid **72** was isolated at 92% yield. The coupling with the aniline derivative was performed in DMF, using *N*,*N*-diisopropylethylamine (DIPEA) as base and HATU, and under US at r.t. for 3 h, the amide **73** was isolated at 56% yield [115]**.**

**Scheme 27:**
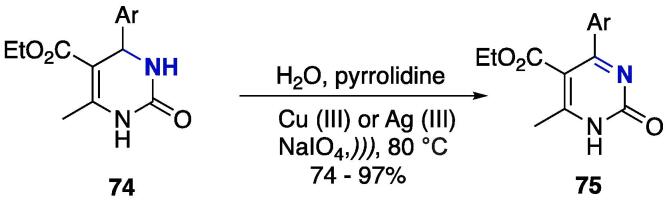
Oxidation of 1,2,3,4-tetrahydropyrimidines **74** using Cu (III) or Ag (III) as oxidizing agents, reported by Gavrilović et al. (2018) [214]**.**

An oxidation protocol based on the oxidation of 1,2,3,4-tetrahydropyrimidines **74** using copper (III) or silver (III) — in the form of (KNa_4_[Cu(HIO_6_)_2_].12H_2_O) and (KNa_4_[Ag(HIO_6_)_2_].12H_2_O) — as oxidating agents was developed under US (Scheme 27) [214]**.** Pyrrolydine was used as a base to abstract the β-hydrogen, and the oxidized products pyrimidin-2(1*H*)-ones **75** were obtained at 74–97% yields, depending on the catalyst applied. In general, Ag (III) provided higher yields than Cu (III) [214].

**Scheme 28:**
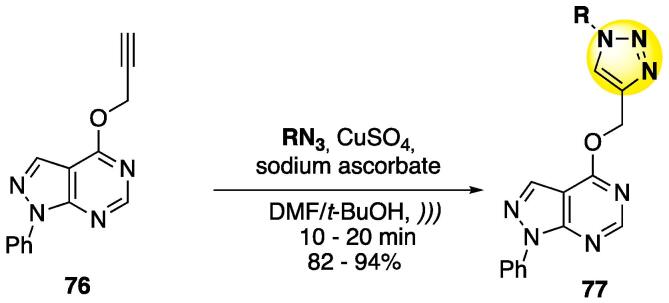
Synthesis of 1,2,3-triazolyl pyrimidine **77** from propargyl-pyrimidine **76**, reported by Kalavadiya et al. (2020) [227].

**Scheme 29:**
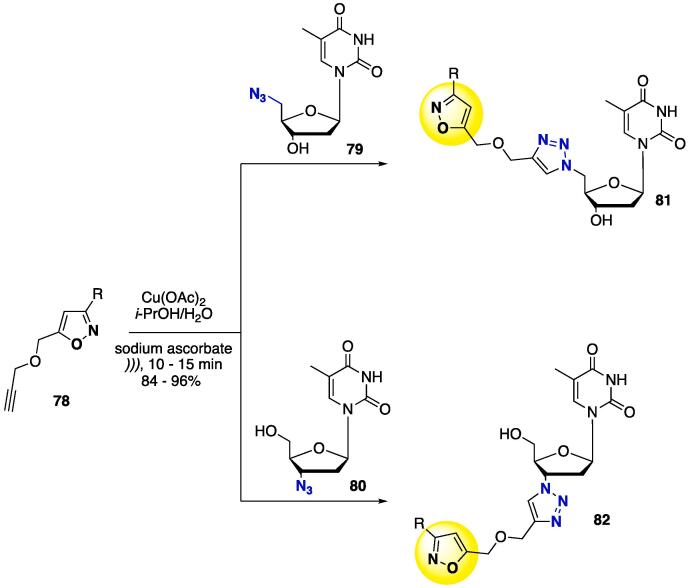
Synthesis of pyrimidine–furan–1,2,3-triazole–isoxazole hybrid molecules **81** and **82**, reported by Zhang et al. (2017) [228].

**Scheme 30:**
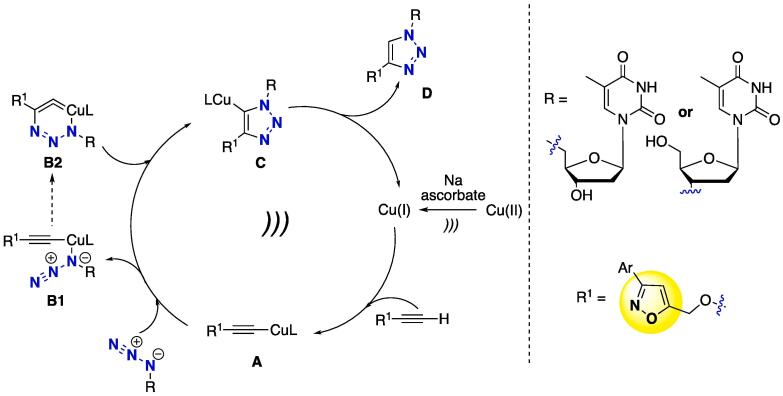
CuAAC catalytic cycle for the US-mediated synthesis of pyrimidine–furan–1,2,3-triazole–isoxazole hybrids, reported by Zhang et al. (2017) [228].

Since the selectivity was observed in the copper-catalyzed azide-alkyne cycloaddition (CuAAC or also known as click reaction) by Sharpless in the early 2000s [215], [216], [217], several known heterocycles have been easily and selectively coupled with the 1,2,3-triazole motif — including pyrimidines [218], [219], [220] — to furnish final scaffolds with enhanced biological and/or pharmacological properties [221], [222], [223]. Given their importance, several US-based methodologies for the synthesis of 1,2,3-triazole-containing molecules have been developed, and a general observation is that they proceed much faster — in most cases the reaction is completed within 20–30 min — than the ones without US irradiation (12–16 h) [224], [225], [226]. Despite the importance of triazole-pyrimidine hybrids, there are two reports related to the construction of the triazole motif in a pyrimidine using US (Scheme 28, Scheme 29, Scheme 30) [227].

The synthesis of 1,2,3-triazole-pyrimidine-pyrazole hybrids **77** was done by using propargyl pyrazolo[3,4-*d*]pyrimidines **76** under common CuAAC conditions (azides, CuSO_4_, and sodium ascorbate as reducing agent) in a DMF/*t*-BuOH mixture as solvent (Scheme 28) [227]. The reaction under conventional conditions took 6 h to complete (84% yield); however, under US, the time decreased to 20 min (94% yield). Seventeen examples were prepared in accordance with this methodology, with 82–94% yields [227].

The synthesis of 1,2,3-triazoles linking oxazole-thymidine-containing scaffolds was assembled by reacting alkyne isoxazole **78** under CuAAC conditions — Cu(OAc)_2_ was used as copper source — with azido thymidines **79** and **80** to furnish 1,2,3-triazoles **81** and **82**, at 84–96% yields (Scheme 29) [228]. The reactions proceeded smoothly at r.t. within 10–15 min (*i*-PrOH/H_2_O as solvent) to provide full conversion of the starting materials, regardless of the less hindered (**79**) or more hindered (**80**) azide source. When done in the absence of US, the reactions took 4–13 h to achieve full conversion (at 45 °C). The authors proposed a mechanism for the click reaction and the key step for the US to catalyze at a faster rate [228]. Scheme 30 shows the catalytic cycle for the US-catalyzed click reaction.

Initially, Cu(OAc)_2_ is reduced *in situ* with sodium ascorbate to furnish the active Cu(I) species, then copper-acetylide **A** is formed and nucleophilic addition of the azide moiety to **A** furnishes the intermediates **B1** and **B2**. After cycloaddition occurred (**B1**), the copper-containing intermediate **C** was obtained, and, after the removal of copper to regenerate Cu(I) in the reaction medium, 1,2,3-triazoles **D** were obtained [216], [229], [230]. In the case of the role of US in this type of reaction, the authors suggested that a higher amount of energy was inserted in the system and US acted in the overall process by enhancing the reaction rate, not only in an isolated step [228].

**Scheme 31:**
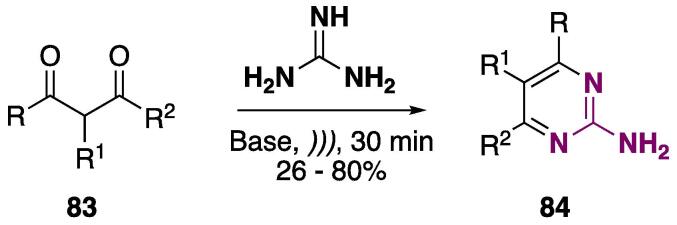
Cyclocondensation reaction between 1,3-diketones **83** and guanidine to furnish 2-aminopyrimidines **84**, reported by Bayramoğlu, Kurtay, and Güllü (2020) [131]**.**

The US-based procedure for the cyclocondensation reaction between 1,3-diketones **83** and guanidine was developed, and 2-aminopyrimidines **84** were obtained at 26–80% yields, depending mostly on the base used in the reaction (NaOH, Na_2_CO_3_, or NaOEt — see Scheme 31). When NaOEt was used, the yields were greatly improved in some cases, especially the ones containing R1 ≠ H [131].

It is important to note that when R or R^2^ in the starting **83** = OEt, hydrolysis of the ester moiety was observed, and (di)hydroxy pyrimidines **84** were obtained in all cases. This is mainly due to the basic nature of guanidine, since parent 1,3-dielectrophiles have been reported to hydrolyze trichloromethyl ketones to furnish their corresponding carboxylic acids [231].

**Scheme 32:**
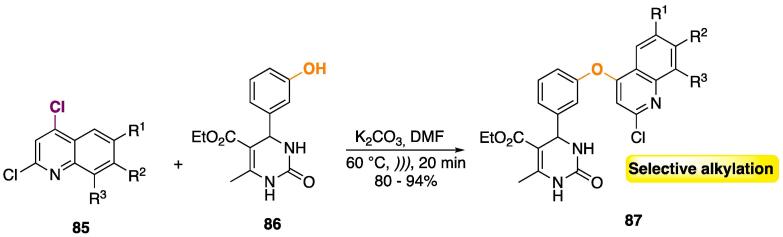
Selective nucleophilic aromatic substitution to furnish pyrimidines bridged with a chloroquine analogue, reported by Balaji et al. (2013) [232].

2,4-Dichlorochloroquines **85** were used as alkylating agents of the phenol moiety of pyrimidines **86** (Scheme 32) [232]. The reaction was conducted in DMF, using K_2_CO_3_ as base. Only the *O*-alkylation of the phenol moiety was obtained, which is a very significant outcome, since the oxygen and both nitrogens of the pyrimidine ring are also nucleophilic (with similar pKa values) and usually furnish regioisomeric products of *N*- or *O*-alkylation [233].

The authors also performed a comparative study of the yields from conventional heating (60 °C, 15 h) and US (60 °C, 20 min) — see Table 8. Remarkably, the yields were significantly higher (up to 21%), and the reaction time decreased from 15 h to 20 min [232].

**Table 8:** Comparative study of the yields of **87** from conventional heating and US irradiation, reported by Balaji et al. (2013) [232].

Entry	R^1^	R^2^	R^3^	Oil bath^a^	Ultrasound^b^
				Yield (%)	Yield (%)
1	Me	H	H	81	94
2	H	Me	H	69	87
3	H	H	Me	73	89
4	OMe	H	H	75	90
5	H	H	OMe	66	87
6	H	Cl	H	73	80
7	Br	H	H	70	84
8	Me	H	Me	78	91
9	2-chlorobenzo[*h*]quinoline			74	85

Reaction conditions: ^a^ DMF, 60 °C, 15 h. ^b^ DMF, 60 °C, 20 min.

**Scheme 33:**
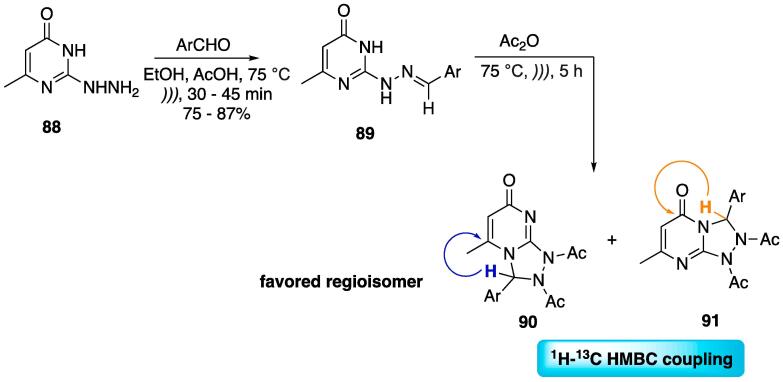
US-mediated synthesis of 1,2,4-triazolo[4,3-*a*]pyrimidines and assignment of the regioisomers obtained by two-dimensional ^1^H–^13^C HMBC NMR, reported by Ashry et al. (2020) [234].

A methodology to prepare 1,2,4-triazolo[4,3-*a*]pyrimidines through a linear sequence was developed (Scheme 33) [234]. Initially, 2-hydrazinopyrimidin-4(3*H*)-one **88** was reacted with aromatic aldehydes (EtOH, AcOH, 75 °C, *)))*, 30–45 min) to furnish benzylidene hydrazones **89** at 75–87% yields. These were cyclized through an acetic anhydride-mediated cyclization double *N*-acetylation strategy to furnish products **90** and **91** as a mixture of isomers (75 °C, *)))*, 5 h), at good overall yields (up to 80%, considering both isomers), with **90** being the one most favored. It is important to note that the isomers could be separated through column chromatography [234].

**Scheme 34:**
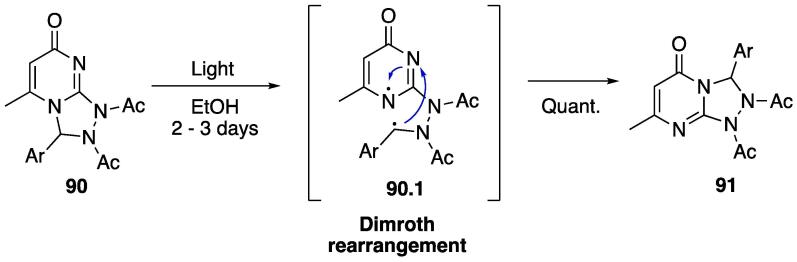
Light-driven Dimroth rearrangement of 1,2,4-triazolo[4,3-*a*]pyrimidines, reported by Ashry et al. (2020) [234].

Although obtaining isomers in this specific type of cyclization reaction is not unexpected when no chiral auxiliaries or selective reagents are used [235], the authors proposed that the Dimroth rearrangement is occurring and converting the obtained **90** into the more stable **91** with the aid of light (Scheme 34). This was confirmed by exposing the pure isolated **90 (**dissolved in EtOH) to light for 2–3 days in order to isolate **91** as a pure compound.

## 5 Derivatizations of the pyrimidine ring

### 5.1 N, O, or S-alkylation(arylation) reactions

The alkylation of pyrimidines is a widely used strategy for achieving important novel physical and bioactive properties of desired compounds [236]. Several alkylating agents have been used; for example, diisopropylzinc for the Soai reaction [237], alkyl sulfonates [238], epoxides [239], ethers [240], and alcohols [241]; however, the one most widely pursued uses alkyl/aryl halides [242], [243], [244], [245], [246]. Even though the aforementioned alkylating agents have been gaining attention in this type of reaction, alkyl halides remain the electrophiles most commonly used for verifying selectivity issues in US-assisted alkylation of pyrimidines, given that alkyl halides usually provide regioisomeric mixtures of *N^1^-*, *N^3^–*, and *S*-/*O*-alkylated products, due to the difficultly in controlling and/or predicting the nucleophilicity of the aforementioned heteroatoms [247], [248].

Table 9 shows the general structures of the starting materials (nucleophiles and electrophiles) used for the *N*-, *S*-, or *O*-alkylation reactions on pyrimidines. It is important to note that the pyrimidine is used as both a nucleophile (entries 1–6, 8, and 9) and electrophile (entries 7 and 10). In most cases, the reactions proceeded smoothly and under regular bimolecular nucleophilic substitution reaction conditions (aprotic polar solvent, base). One can easily see that, in general, only alkyl chlorides were used (with the exception of entries 2 and 3, in which methyl iodide was used), which indicates that the reaction was feasible using this poor leaving group (chloride) and was able to be carried out in the absence of heating and yet furnishing good isolated yields and short reaction times in most cases.

**Table 9:** General structures of starting materials and products, as well as reaction conditions and isolated yields from alkylation/arylation reactions in pyrimidines.

**Scheme 35:**
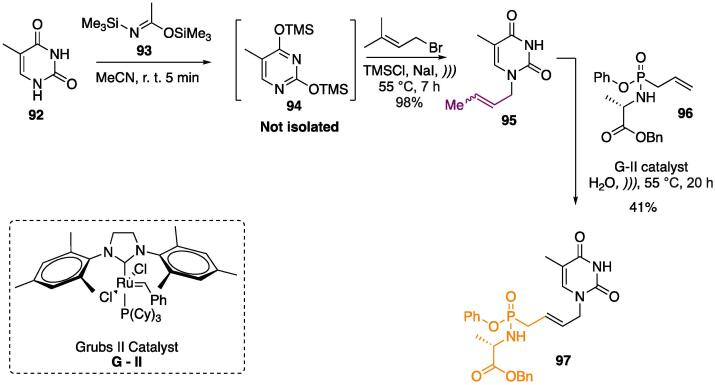
US-assisted protection, selective *N*-alkylation, and metathesis of pyrimidines **92**, reported by Bessieres et al. (2018) [259].

A very well explored protocol for selectively introducing alkyl substituents at *N^1^*- into uracyl derivatives is the initial double *O*-alkylation with a source of trimethylsilyl group [255], [256], [257], [258] (in this work, [259] *bis*-trimethylsilylacetamide) furnishing *O^2^–* and *O^4^*-TMS pyrimidine **94** at quantitative yields. Further reaction with crotyl bromide (under US) furnishes only *N^1^*-substituted uracyl **95** (the other nitrogen is too hindered by the TMS moiety to act as a nucleophile) as a mixture of (*E*) and (*Z*)-isomers, at 98% yield (Scheme 35).

In a second part of the work (Scheme 35), the authors used the ruthenium-based catalyst Grubbs II (G-II) — which has been widely used in the metathesis of several complex molecules [260], [261], [262], [263] — in a strategy combining it with US for preparing alkyl phosphonates **97** (Scheme 35). During this stage, the authors observed that other catalysts (Hoveyda-Grubbs II and Zhan catalyst-1B) were not effective in promoting the formation of **97**. When G-II was used in the absence of US (using conventional heating) or in a solvent other than H_2_O (CH_2_Cl_2_), no formation of the product was observed, thus demonstrating the power of US in catalyzing this metathesis reaction in *N*-alkylated pyrimidines [259].

### 5.2 Other amino derivatizations in 2-aminopyrimidines

**Scheme 36:**
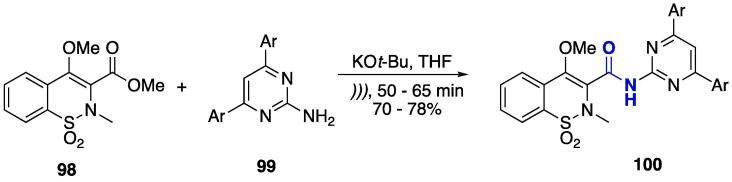
Synthesis of benzothiazine–pyrimidine hybrids bridged by an amide bond, reported by Tamatam et al. (2019) [264].

The amino group at the 2-position of the pyrimidine ring allows other derivatizations such as the preparation of amides. The aminolysis of benzothiazine 3-carboxylate **98** was done using 2-aminopyrimidines **99** (Scheme 36). The reaction was conducted in the presence of potassium *tert*-butoxide in THF (r.t., *)))*, 50–65 min). The corresponding carboxamides were obtained at 70–78% yields [264].

**Scheme 37:**
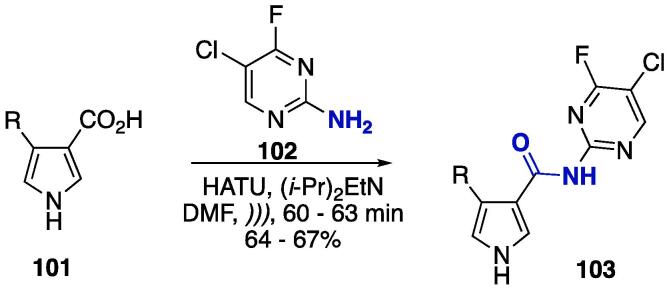
US/HATU-catalyzed carboxylic acid and amine coupling to furnish 1*H*-pyrrole pyrimidine hybrids bridged by an amide bond, reported by Syamaiah et al. (2014) [265].

In a very similar protocol, the preparation of carboxamides was done using 1*H*-pyrrole 3-carboxylic acids **101** and 2-amino-4-fluoro-5-chloropyrimidine **102**, and HATU as the coupling agent (Scheme 37) [265], with the resulting carboxamides **103** obtained at moderate yields (64–67%).

**Scheme 38:**
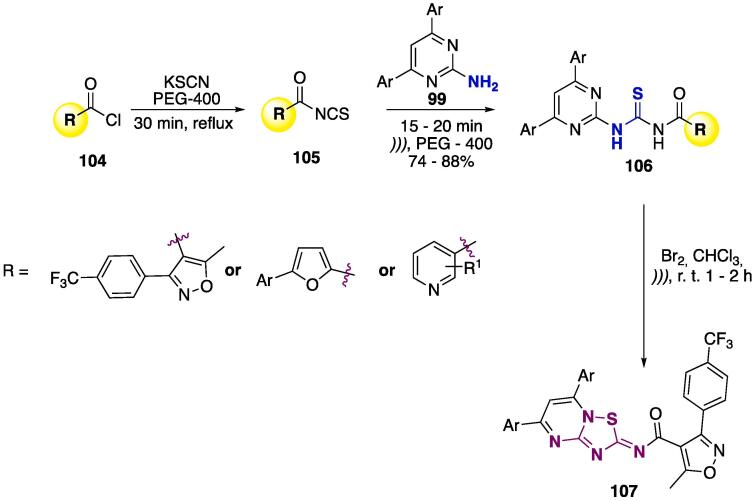
US-assisted synthesis of thioureas derivatives **106**, and further cyclization mediated by Br_2_, reported by Xue et al. (2004) [266], Ke and Cao (2011) [267], and Jiang et al. (2012) [268].

The 2-amino moiety was also reacted with isothiocyanates **105** (previously prepared from acylchlorides **104** and KSCN), using PEG-400 as solvent, to furnish products **106** at good yields (74–88%) and in short reaction times (15–20 min) — see Scheme 38 [266], [267], [268]. A very interesting transformation of **106** was performed by reacting with molecular bromine (CHCl_3_, *)))*, r.t., 1–2 h, Scheme 38) to furnish 2*H*-1,2,4-thiadiazolo[2,3-*a*]pyrimidines **107** at good yields (up to 80%) [268].

**Scheme 39:**
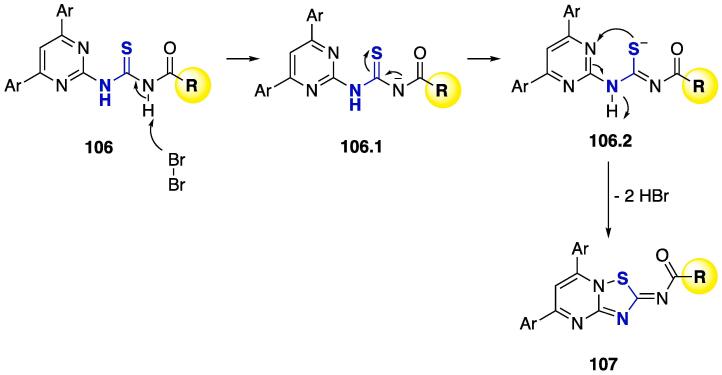
Mechanism proposed for the synthesis of 2*H*-1,2,4-thiadiazolo[2,3-*a*]pyrimidines **107**, reported by Jiang et al. (2012) [268].

A mechanism was proposed for the cyclization of **106** mediated by Br_2_ (Scheme 39). Initially, bromine deprotonates the NH and, through charge delocalization (**106.1**), the thiol moiety is formed and attacks the pyrimidinic nitrogen (**106.2**), thus furnishing cyclic 2*H*-1,2,4-thiadiazolo[2,3-*a*] pyrimidines **107** and the elimination of two HBr molecules (Table 8, Table 9).

## 6 Conclusions and outlook

A concise and thorough summary of the achievements in the US-assisted synthesis and derivatization of pyrimidine scaffolds was presented. In recent years, US has emerged as an alternative source of energy in the synthesis of this targeted heterocycle. Among its main advantages, the following can be mentioned: reduction in reaction times, higher yields, fewer reaction steps, and less generation of byproducts than conventional methods. The role of US as a reaction accelerator, its synergic effects when combined with other additives (catalysts, ILs, etc.) and the benefits of the reactions (e.g., selectivity issues) were also discussed. We hope that the information gathered herein inspires and supports future synthetic researchers looking for efficient methods to prepare this highly biologically relevant scaffold, and to improve upon the methods already known and apply them to less explored protocols.

## CRediT authorship contribution statement

**Mateus Mittersteiner:** Conceptualization, Methodology, Investigation, Writing - original draft, Writing - review & editing. **Fellipe F. S. Farias:** Methodology, Investigation, Data curation, Visualization, Writing - original draft. **Helio G. Bonacorso:** Resources, Project administration, Funding acquisition. **Marcos A. P. Martins:** Resources, Project administration, Funding acquisition, Supervision. **Nilo Zanatta:** Conceptualization, Methodology, Investigation, Writing - original draft, Writing - review & editing, Supervision, Resources, Project administration, Funding acquisition.

## Declaration of Competing Interest

The authors declare that they have no known competing financial interests or personal relationships that could have appeared to influence the work reported in this paper.
